# The Effects of a 6-Week Swimming Intervention on Gross Motor Development in Primary School Children

**DOI:** 10.3390/children11010001

**Published:** 2023-12-19

**Authors:** Nicole A. Pratt, Michael J. Duncan, Samuel W. Oxford

**Affiliations:** 1 Department of Health and Life Sciences, Coventry University, Coventry, CV1 5FB, UK; drnicoleannprattmscbschons@outlook.com (N.A.P.); apx327@coventry.ac.uk (S.W.O.); 2 Centre for Sport, Exercise and Life Sciences, Coventry University, Coventry CV1 5FB, UK

**Keywords:** swimming, aquatic, motor competence, fundamental movement, dryland, physical activity, physical education

## Abstract

(1) Background: This study examines the effects of a 6-week swimming intervention on motor competence in children. (2) Methods: A total of 107 children (*n* = 52 boys, *n* = 55 girls) aged 7.8 ± 0.63 years that were recruited from five primary schools in central England participated in this study, undertaking either an aquatic intervention once a week for six weeks or acting as a control group completing their usual physical education program. Participants underwent pre- and post-assessments of general motor competence using the Test of Gross Motor Development, Third Edition (TGMD-3) (a process measure) and a composite of 10 m running sprint time and standing long jump distance (product measures). Aquatic motor competence was assessed via the Aquatic Movement Protocol (AMP). Fear of drowning and swimming opportunities were also assessed by implementing a questionnaire. (3) Results: Following a mixed-model ANOVA, an overall main effect was found from pre (40.05 ± 13.6) to post (48.3 ± 18.6) for TGMD-3 scores (*p* < 0.05) and pre (38.7 ± 31.7) to post (50.6 ± 36.8) for AMP scores (*p* = 0.001). A negative significant relationship was found between AMP scores with both fear of water (*p* = 0.01) and fear of drowning (*p* < 0.05). A positive significant relationship was found between swimming opportunities and AMP score (*p* = 0.001). (4) Conclusions: The aquatic-based intervention improves not only aquatic motor competence but also transfers improvements in dryland movement competencies. Future research should look to implement control groupings which do not participate in swimming to further investigate the difference between swimmers and non-swimmers; however, due to swimming being a part of the national curriculum in England, this may not be feasible.

## 1. Introduction

Fundamental movement skills (FMS) are considered the building blocks of complex movements required for participation in sports and physical activities [1]. FMS consists of two categories locomotor skills (movements which transfer the body from one location to another) and object control skills (transferring, propelling or catching objects). Performing object control and locomotor skills requires the activation of large muscle groups [2]. Physical literacy (PL) is an important component of a child’s primary education and development, with FMS being an underpinning element of PL, forming a part of the national curriculum [3,4]. Physical literacy is defined as “motivation, confidence, physical competence, knowledge and understanding to value and take responsibility for engagement in physical activities for life” [5]. Improvements in FMS, either as a part of PL development or in isolation, have become prominent in PE curricular globally [6]. FMS proficiency is a crucial aspect in achieving and retaining physical activity (PA), a healthy weight and fitness and developing complex motor skills to be used in current and later life [7,8]. Therefore, it is found that children with higher FMS proficiency are more likely to partake in PA [8,9], obtain higher fitness levels [10] and are less likely to be overweight [10,11]. Stodden et al. [3] designed a model which outlines that in middle to late childhood, FMS development plays a vital role in weight status through PE, directly impacting PA.

In England, the national curriculum for PE highlights the development of a broad range of FMS, including running, jumping, throwing and catching in both isolation and in combinations [6]. There is a concern that FMS competencies are poor worldwide; therefore, there has been a call for effective interventions to be implemented to focus on FMS development [12,13,14,15]. Due to the compelling evidence FMS has on a child’s motor development (the change in behaviour over a lifetime and the processes which these changes underlie [16]), a variety of interventions have been trialled in school children to enhance FMS [17,18,19]. These prior interventions had success but focus on FMS practice in isolation, without the context of sports performance [20]. The PE curriculum in England emphasises a transition of FMS into sporting skills; therefore, providing a sport-related FMS intervention would be more pragmatic and aligned with the curriculum [20]. One established intervention takes this into account, investigating the effects of the Badminton World Federation (BWF) shuttle time on both product and process assessments of FMS in children. Duncan et al. [20] provided a program which aimed to achieve primary PE objectives through a badminton-based intervention in children aged between 6 and 11 years. Duncan et al. [20] had two groupings within this study, a shuttle time intervention group and a control group which undertook their normal routine PE lessons. Both process and product measurements of FMS were employed; process FMS was assessed through the Test of Gross Motor Development, Second Edition (TGMD-2) and product FMS incorporated the assessment of 10 m sprint time, standing long jump and a 1 kg seated ball throw [20]. The shuttle time program was based upon exercises specified by the BWF consisting of a warmup and a main body [20]. The intervention incorporated the development of balance, coordination, underhand throwing, catching, striking, running, jumping and correct use of a racquet (to grip and swing—badminton). Duncan et al. [10] found that the key aspect of a shuttle time program is that the developments in FMS competence through badminton are applicable to a variety of sports. This aligned with prior research, suggesting that school-based interventions significantly enhancing both the process [20,21] and product assessment of FMS [16,20].

FMS-focused interventions among children should employ coaching cues, attentional focus instructions with plyometric-type exercises and a physical intervention to have maximum success rates in motor development [22]. Interventions should focus on mechanical efficiency, including the biomechanical principles of action [22]. Studies incorporated within a review article by McDonough, Liu and Gao [23] targeting FMS have been shown to significantly increase FMS competence in both adolescents and children. Previous research highlighted interventions designed to target motor development ranged between 4 weeks and 6 months [23]. Previous research focused on interventions to develop FMS incorporated a range of lesson and intervention durations [16]. Logan et al. [16] implemented a meta-analysis that investigated a large volume of papers focusing on different durations of interventions and lesson lengths and found a non-significant relationship between these variables. The National Association for Sport and Physical Education (NASPE) recommends that preschool-aged children should engage in a minimal duration of 60 min of PA per day [24]. More recently, the UK government has advised that children should participate in at least 2 h of PE per week. Hardman and Logan et al. [16,25] conducted a meta-analysis and found no significant relationship between intervention duration and FMS improvements. Duncan et al. [20] found that interventions of a frequency of once a week in primary-aged children may not be effective due to movement patterns becoming fully developed at this timescale.

To fully understand which interventions develop FMS more effectively, it is important to track development through FMS assessment methods. There is a magnitude of protocols that can be used to assess FMS, including both product- and process-based assessments. Therefore, selection is dependent on equipment, time, information required and the population of interest. Process-based assessments are the most commonly used within the literature, specifically those targeting FMS competency, with the Test of Gross Motor Development (2nd or 3rd editions) by Ulrich [2,26] being the most commonly used FMS assessments for young children.

Swimming was introduced into the national curriculum in 1994 due to the profound number of benefits it has on a child’s lifespan [27]. Swimming has evolved massively since the first document written on swimming in 1538. Today, the term swimming is defined as a sport in which one generates forces by use of the limbs and body movements to overcome the resistance that the water presents [28]. Participating in swimming has been seen to promote health-related benefits, including social, emotional and psychological well-being, cardiorespiratory fitness, flexibility, endurance, aerobic capacity, muscle mass, body composition and quality of life [29,30]. The national curriculum of England states that a primary-aged graduate must be able to swim competently, confidently and proficiently over a minimum distance of 25 m, incorporating a range of strokes [30], and to be able to perform safe self-rescue in a range of water-based situations [30]. Previous research in the aquatic environment indicates the importance of introducing swimming PE programs at primary ages, not only as a safety aspect but to provide advancements in motor competence. Motor competence is defined as a person’s ability to perform a range of motor acts, including movement coordination and control—an outcome that is needed for daily tasks [31].

Martins et al. [32] found that children who participate in regular swimming lessons have better general motor competence, indicating improvements in aquatic skills and an increase in motor development on dryland. Martins et al. [32] divided children into groups in relation to previous swimming experience, those with and without swimming experience, by incorporating the TGDM-2 and analysing 12 specific skills on this FMS assessment [32] to see the impact swimming experience has on a child’s FMS. This aligns with Rocha et al. [21], who assessed FMS through the TGMD-2 assessment at three time points; at baseline and at 5, 10 and 30 months of swimming or soccer practice. The soccer and swimming practice took place at the same time with swimming following Langendorfer and Bruya’s [33] aquatic readiness program, and soccer taking place on a synthetic outdoor sports field following a similar model by Bunker and Thorpe [34]. This enabled them to investigate the effects that swimming and soccer performance had on motor development [32]. Rocha et al. [21] found that children who participated in swimming lessons within an educational setting (school swimming lessons) had more defined motor development on a range of FMS tests. Swimming practitioners have reported that young children who participate in swimming have superior and continuous motor development, especially within the object control domain [35]. However, FMS interventions within a school setting are lacking in attention across all aspects of the national curriculum in PE, with a particular absence in the research around the aquatic environment.

A meta-analysis identified childhood as an important timeframe to target FMS development; therefore, implementing interventions during this period is crucial [36]. Currently, to date, most FMS research is on dryland movements/sports, and those in the aquatic environment are missing a vital element of assessment in aquatic motor competence. Consequently, the contrasting environments need to be investigated to see what effects they have on FMS development. Current aquatic interventions have primarily focussed on special populations, with many studies being aimed at children with cerebral palsy and disabilities, due to swimming being a gentle impact sport [12]. Cole and Becker [37] found that water-based interventions improve muscle strength, lung function, balance, coordination and posture. Berukoff and Hill [38] found that water provided opportunities that cannot be obtained on land, including improvements in physiological and psychological achievements for individuals with disabilities. This is due to the nature of the environment: the warm water reduces muscle tone, which allows for individuals with high muscle tone to move and perform more freely in the water [39]. Although there is a handful of literature on special populations, interventions in abled-bodied children are scarce. Therefore, through previous findings, it is clear that the research is focused upon clinical populations; hence, future research needs to be directed towards typically developing children at a primary age.

Swimming is seen to promote superior motor competence, as a whole, through the direct links it has with FMS movements on land and with the added benefit of being in the aquatic environment [33]; however, there are other crucial factors that can also have an impact a child’s ability in the water. Irwin et al. [36] found fear of drowning to be a strong predictor of swimming performance/competence, with fear around the water environment being a considerable barrier for individuals learning to swim. This results in a negative feedback loop of individuals who have lower swimming competence being put at a higher risk of fatal and non-fatal drowning [40]. Due to the negative impact that fear of water and drowning has on an individual’s ability/participation in swimming, it is important to assess these factors to investigate its relationship with motor competence. Previous aquatic experiences are a crucial factor in learning to swim due to the development of swimming self-efficacy [41]. Self-efficacy refers to one’s judgment on their capability to successfully perform specific tasks at given levels [42]. Bandura’s [43] self-efficacy theory suggests that efficacy plays a vital role in a predictive and mediational role in one’s thought patterns, motivations and behaviour. Berukoff et al. [38] found those with low self-efficacy had a lack of swimming opportunities; therefore, this directly impacts their fear of drowning and water due to the environment being foreign.

The development of the AMP assessment method has allowed researchers to investigate the impact swimming competence has on dryland motor competence [44]. The AMP consisted of eleven aquatic motor skills, including the following three strokes—front crawl, back crawl and breaststroke—and 8 aquatic skills—push and glides, log rolls, sculling feet first and headfirst, treading water, entering water via a jump, submersion, floating and tuck position. All of these are the main skills concentrated on the national curriculum in England and Swim England’s strategy [swim England]. Pratt et al. [44] implemented the TGMD-2 for the assessment of FMS and used this to make direct comparisons between FMS and aquatic motor competence (AMP). In developing this assessment method, Pratt et al. [45] were able to investigate the impact swimming competence has on dryland motor competence. Pratt et al. [44] found that children with lower FMS achieved lower competence scores within the aquatic environment, with individuals classified as superior in FMS on land performing significantly better within the water. This highlights a clear link between FMS (TGMD-2) and aquatic movement skills (AMP). The aforementioned interventions targeting FMS development are based on dryland, leaving the aquatic environment undetermined, thus leaving a major section of the national curriculum uninvestigated. Therefore, implementing an aquatic intervention as a means to target FMS development will fill a major gap in the research. Due to the outlined importance of implementing interventions in the research on dryland and with the growing evidence of the impact swimming has on motor development, it is essential aquatic interventions are developed [21,33,44]. An aquatic intervention can further support the previous findings and investigate the link between dryland and aquatic motor competencies through an interventional approach. For this to be actioned, an assessment of aquatic motor competence needs to be implemented; to date, the Aquatic Movement Protocol (AMP) is the only method for assessing aquatic motor competence. Thus, the AMP will be implemented within this study. Consequently, this study aims to develop an aquatic motor competence intervention to target general motor competence on dryland, aiming to explore the links between general motor competence through FMS assessment with TGMD-3 and aquatic motor competence through AMP, including an investigation on the impacts that swimming opportunities, fear of water and drowning have on a child’s ability to perform in the aquatic environment.

## 2. Materials and Methods

### 2.1. Participants

A convivence sample of (*n* = 5) primary schools that were part of a swimming program through CVLife (a charitable trust, providing a range of sporting, social, education and recreational activities to underrepresented groups) from the Coventry Area agreed to be part of this study. Following ethics approval (*p* = 61,595), parental informed consent and participant consent was obtained for 107 children (*n* = 52 boys, *n* = 55 girls) in school years 3, 4 and 5 (7.8 ± 0.63 years of age). Gatekeeper consent was also collected from CVLife and participating schools. Inclusion criteria included any children either in years 3, 4 or 5 and as part of any schools who were signed up as a part of CVLife school swimming lessons. Inclusion included children who have not registered with special educational needs or any form of musculoskeletal issue that could impair movement.

### 2.2. Study Design

This study employed a cluster randomised intervention design. Five individual classes from five different schools were allocated in either of two conditions: intervention (aquatic intervention) or control group. Each class from the schools was subsequently drawn at random and split into two groups—intervention (*n* = 53) (*n* = 23 girls, *n* = 29 boys) and control (*n* = 54) (*n* = 30 girls, *n* = 24 boys)—both groupings were selected by chance (coin toss). Data collection sessions were completed at either the primary schools themselves or at their local swimming pool hosted by CVLife. Dryland testing was conducted within the primary schools’ sports halls, all of which were similar in size and had replicated equipment/station setups per primary school. Aquatic testing took part at two swimming pools which had the same dimensions, equipment setups and sections for four classes. Setups were replicated across each school with children being split into sections of the pool dependent on their swimming ability as per health and safety guidelines outlined by CVLife. Swimming instructors were allocated to each group within the pool and were kept the same throughout the entirety of this study. Prior to and immediately following the 6-week intervention, participants in both groups were assessed on measures of the process and product assessment of FMS as well as aquatic motor competence [20]. A 6-week intervention was selected to eliminate any gaps between pre/post-intervention testing with a total of an 8-week testing timescale which fitted within one school term.

#### 2.2.1. Control Group

The control groups attended two standard 60 min physical education classes, one of which was delivered by their usual PE teacher and consisted of a range of sports, including football, dance, rounders, badminton and gymnastics, across the five schools. The second standard PE class consisted of a structured swimming lesson delivered by trained swimming instructors hired through CVLife.

#### 2.2.2. Intervention Group

**Figure 1:**
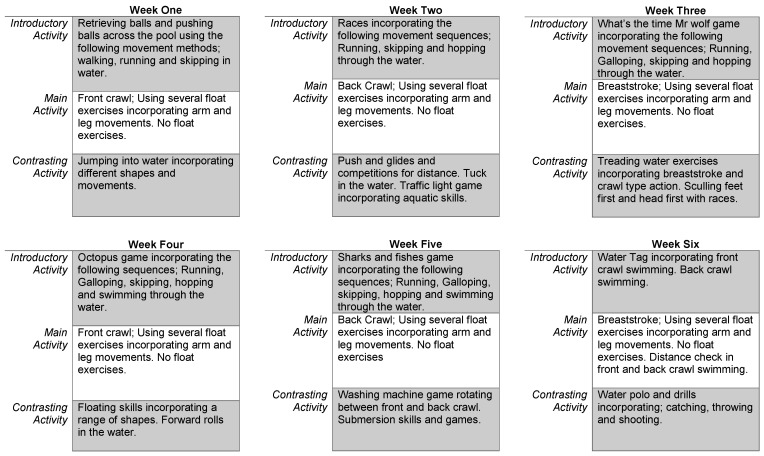
Detailed focus of six-week aquatic intervention.

The intervention groups undertook an aquatic program over a six-week period in place of one (out of two) statutory physical education sessions. A six-week progressive aquatic motor competence program, based on the learn to swim program consisted of an introductory activity (5–10 min), main activity (20–25 min) and a contrasting activity (5–10 min) per session (Figure 1), dependent on whether they were scheduled for a 30 or 45 min lesson [36]. The intervention focused on the development of three aquatic strokes—front crawl, back crawl and breaststroke—and eight aquatic skills—push and glides, log rolls, sculling feet first and headfirst, treading water, jumping into the water (in various shapes), submersion, floating (in various shapes) and tuck. The design of the intervention differed from typical swimming lessons as it was focused on enhancing motor competence on dryland by implementing land-based movements within the water. This was to replicate movements on dryland with the added benefit of water resistance. The intervention itself and the strokes and skills it entailed were included in the AMP assessment. Therefore, the intervention focused on improving skills which would be assessed via the AMP.

The intervention group undertook a 60 min second weekly physical education lesson during the intervention period, which was focused on their standard school curriculum’s sport or activity. The investigator oversaw all pool-based intervention sessions which were delivered by the same qualified swimming instructors throughout. Any child who missed more than one aquatic session in the intervention period was removed from the final analysis. During the intervention, participants received continuous feedback of the quality of each movement in accordance with [20,46].

### 2.3. Anthropometry

Anthropometric data, including height (cm) and sitting height (cm), were recorded to the nearest cm using a stadiometer (SECA Instruments Ltd., Hamburg, Germany); mass (kg) and body fat (%) were recorded using electronic scales (TANITA scales C-300, Tokyo, Japan).

### 2.4. General Motor Competence

Process motor competence was analysed using eleven FMS, with 6 being object control skills consisting of bounce, overhand throw, roll, catch, strike and kick, and 5 locomotor skills consisting of gallop run, skip, jump and hop—all from the Test of Gross Motor Development, Third Edition (TGMD-3) [30]. During this, the identification of whether each aspect of the skill was either absent or present in each participant was carried out. TGMD-3 assessments took place in the sports hall of each primary school, which was divided into stations per skill for recording. A total of three attempts of each skill were recorded, with two being scored and one being as a practice. Scores from the two recorded trials were totalled to obtain a raw score for each skill. Scores for all skills were then totalled to create a total general motor competence score (scored 0–74). Scores from run, jump, hop, skip and gallop were totalled to calculate a raw score for locomotor skills (scored 0–26). Scores from the overhand kick, strike, throw, roll and bounce were calculated to create a raw score for object control skills (0–48), following recommendations of administrating the TGMD-3 [26]. Each skill was recorded using a Nikon B500 video camera, (New Delhi, India), with each skill being evaluated using Quintic Consultancy Ltd., Coventry, UK biomechanics analysis software V26. Video clips of each individual skill were uploaded to Quintic and evaluated in slow motion to capture all efficiencies and deficiencies. Three trials of each skill were recorded with the first as a practice, and the second and third were scored. Subjects’ scores were calculated for locomotor competence (0–26) and object control motor competence (0–48). An experienced researcher assessed the children’s movement through analysis of the videos and scored as per guidelines recommended by Ulrich [26], according to recommended guidelines for scoring of the TGMD-23 [10]. During the evaluations, the videos’ inter- and intra-rater reliabilities (ICC), in comparison to an expert, were calculated to ensure that there were no more than 10% discrepancies between each skill with no more than (≥80% agreement) difference in scores according to [46]. Process motor competence was analysed in accordance with [47], consisting of both standard long jump distance and 10 m sprint time.

Product measures were also recorded in the sports hall located of each primary school that partook in this study. A 10-metre sprint (s) was timed using Smart Speed gates (Fusion Sport, Coopers Plains, Brisbane, Australia). Standing long jump (cm) was measured with a long tape measure to measure the distance covered as per landing. Scores from two recorded trials were totalled to obtain a raw score for each skill and again one counted as a practice trial. During the analysis, composite scores of product motor competence were evaluated through developing a z score for each skill, with a composite score as a total of both z scores.

### 2.5. Aquatic Motor Competence

Aquatic motor competence was recorded in accordance with [44] assessing aquatic skills and strokes. Aquatic skills were split into components consisting between 3 and 6 components per skill. Similarly to TGMD-2 and TGMD-3, the AMP identifies whether each aspect of the skill was either absent or present in each participant. Aquatic motor competence was analysed using eleven fundamental aquatic skills, with 3 being aquatic strokes—breaststroke, front crawl and back crawl—and 8 being aquatic skills—floating, log roll, submersion, jump, sculling feet first, and sculling headfirst, treading water and tuck. Each skill was recorded using a Nikon B500 video camera, New Delhi, India with each skill being evaluated using Quintic Consultancy Ltd., Coventry, UK biomechanics analysis software V26. Each skill was uploaded to Quintic and evaluated in slow motion to capture all efficiencies and deficiencies in the water. Two of the three trails were evaluated to calculate a score for aquatic motor competence (0–138). Scores from log roll, jump, treading water, floating, tuck in water, submersion, sculling feet first and sculling headfirst skills were totalled to create a raw score for aquatic skills (0–72). Scores from front crawl, back crawl and breaststroke were totalled to create a total score for aquatic strokes (0–66). AMP assessments took place at a swimming pool within CVLife where children performed each skill within a section of the pool dependent on their swimming ability. During the evaluations, the ICCs of the videos, in comparison to an expert, were calculated to ensure there were no more than 10% discrepancies between each skill with no more than (≥80% agreement) difference in scores according to [44].

### 2.6. Fear of Drowning and Swimming Opportunities

The fear of drowning and swimming opportunities questionnaire was implemented in accordance with [40] to assess the level of fear an individual has towards water and drowning, including assessing the number of aquatic opportunities of each participant.

### 2.7. Statistical Analysis

IBM SPSS Statistics for Windows (Version 24.0. Armonk, NY, USA: IBM Corp) was used to undertake all statistical analysis. Data were initially assessed objectively to ensure normal distribution with the use of the Shapiro–Wilk test. A two (pre and post) by two (intervention and control group) mixed-model ANOVA was used to analyse any main effects, comprising pre to post effects by group interactions for general motor competence, aquatic motor competence and sex. This was completed for total scores (TGMD-3 and AMP), subcategories (locomotor, object control, aquatic skills and strokes) and each individual skill. Variances were made equal by implementing Levine’s tests of equal variances. A repeated measures analysis of covariance and repeated measures ANOVA with Greenhouse–Geisser correction was implemented to compare means across multiple variables. Bivariate Pearson correlations were used to examine the relations between data sets. Where variances were unequal, *p* value was implemented as *p* < 0.001. Where significant interactions were highlighted, paired sample *t*-tests were conducted on the variable to identify if each group (control and intervention) had significantly increased. Significance was set at *p* < 0.05.

## 3. Results

Repeated measures analysis of covariance indicates height, mass, and body fat had no significant (*p* < 0.05) influence on pre to post TGMD-3 or AMP scores.

**Figure 2:**
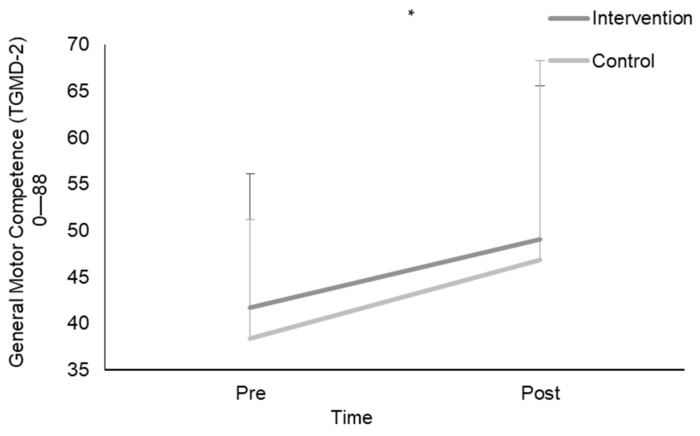
Mean TGMD-3 scores pre and post 6-week aquatic intervention for both intervention and control groupings. * Significant differences between pre to post general motor competence scores.

Following a two (pre and post) by two (intervention and control) mixed-model ANOVA, there was an overall main effect from pre (40.05 ± 13.6) to post (48.3 ± 18.6) for TGMD-3 scores (*p* < 0.05) (Figure 2). Related results were found for both locomotor and object control skills with an overall main effect for locomotor from pre (22.4 ± 6.5) to post (26.6 ± 8.9) and object control pre (18.5 ± 8.2) to post (22.2 ± 12.3) (*p* = 0.001). No sex interactions were identified for TGMD-3, locomotor and object control skill scores (*p* > 0.05).

**Figure 3:**
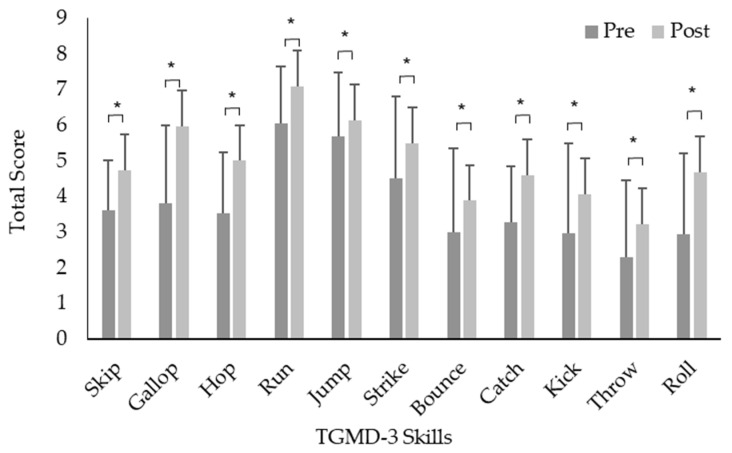
Mean ± SD Test of Gross Motor Development, Second Edition (TGMD-3) scores for all subcategories both pre- and post-intervention. * Significant differences between pre- to post-skill score.

A repeated measures ANOVA with the Greenhouse–Geisser correction determined that the mean TGMD-3 scores for the intervention groups significantly increased between pre (41.7 ± 14.4) to post (48.9 ± 16.5) time points (F (1,50) = 9.165, *p* = 0.004) (Figure 2). The control groups significantly increased between pre (38.5 ± 12.7) to post (47.7 ± 20.6) time points (F (1,52) = 8.641, *p* = 0.005) (Figure 2). Locomotor skills for intervention groups significantly increased between pre (22.3 ± 6.5) to post (27.4 ± 7.2) time points (F (1,49) = 22.362, *p* = 0.001) (Figure 3, Table 1). The control groups significantly increased between pre (22.4 ± 6.6) to post (25.9 ± 10.3) time points (F (1,51) = 4.372, *p* = 0.042) (Figure 3, Table 1). Object control skills for intervention groups increased between pre (20.2 ± 8.6) to post (22.98 ± 10.8) time points, however, not significantly (F (1,48) = 3.358, *p* > 0.05) (Figure 3, Table 1). The control groups significantly increased between pre (16.8 ± 7.6) to post (21.5 ± 13.6) time points (F (1,51) = 6.732, *p* = 0.012) (Figure 3, Table 1).

The mixed-model ANOVA showed a significant main effect pre- to post-data for ten out of the eleven skills in girls (*p* < 0.05), with the FMS skill jump being non-significant (*p* > 0.05) (Table 1). A significant main effect was found for pre- to post-data for boys for ten out of the eleven FMS (*p* < 0.05), with the FMS skill jump and bounce being non-significant (*p* > 0.05) (Table 1). A main effect was found for both time points (pre to post) and interventions (control and intervention groups) for girls for the FMS skill skip (*p* < 0.05) (Table 1). A main effect was found for both time points (pre to post) and interventions (control and intervention groups) for boys for three out of the ten FMS skills, comprising the skip, hop and throw (*p* < 0.05) (Table 1).

The 10 m sprint best time was found to be significant for both boys and girls for both pre- and post-data collection (*p* < 0.05); however, it was non-significant between the control and intervention groups (*p* > 0.05). Jump distance for both boys and girls was found to be non-significant for both time points (pre and post) and interventions (control and intervention groups) (*p* > 0.05).

Following a two (pre and post) by two (intervention and control) mixed-model ANOVA there was an overall main effect from pre (38.7 ± 31.7) to post (50.6 ± 36.8) for AMP scores (*p* = 0.001). Equivalent results emerged for both aquatic strokes pre (20.2 ± 18.9) to post (26.7 ± 20.9) and aquatic skills pre (19.0 ± 13.5) to post (24.5 ± 17.3) (*p* < 0.05). No sex interactions were identified for AMP, aquatic strokes and aquatic skill scores (*p* > 0.05).

**Figure 4:**
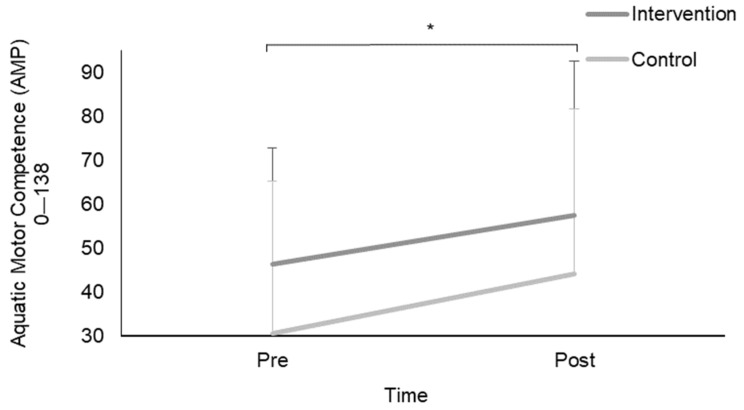
Mean AMP scores pre and post 6-week aquatic intervention for both intervention and control groupings. * Significant differences between pre to post aquatic motor competence scores.

**Figure 5:**
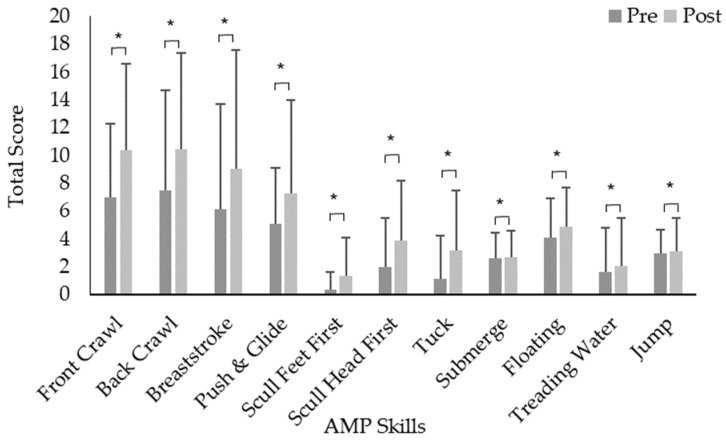
Aquatic Movement Protocol (AMP) scores for all subcategories both pre- and post-intervention. * Significant differences between pre- to post-score.

Repeated measures ANOVA with the Greenhouse–Geisser correction also determined that mean AMP scores for intervention groups significantly differed between pre (47.3 ± 25.9) to post (57.6 ± 34.9) time points (F (1,49) = 6.274, *p* = 0.016) (Figure 4). Control groups significantly differed between pre (30.7 ± 34.5) to post (44.1 ± 37.5) time points (F (1,53) = 15.596, *p* < 0.05). Aquatic strokes for the intervention groups significantly differed between pre (26.1 ± 16.6) to post (32.7 ± 20.6) time points (F (1,49) =6.740, *p* = 0.012) (Figure 5, Table 1). Control groups significantly differed between pre (14.5 ± 19.4) to post (21.04 ± 19.8) time points (F (1,52) = 11.519, *p* = 0.001) (Figure 5, Table 1). Aquatic skills for the intervention groups significantly differed between pre (21.6 ± 10.2) to post (25.1 ± 15.3) time points (F (1,48) =4.404, *p* = 0.041) (Figure 5, Table 1). Control groups significantly differed between pre (21.6 ± 1.5) to post (25.1 ± 2.2) time points (F (1,52) = 16.051, *p* < 0.05) (Figure 5, Table 1).

Bivariate correlations indicate no significant differences between swimming opportunities and fear of drowning questionnaire with AMP, aquatic strokes, and aquatic skills pre and post total scores. Correlations show fear of water to be significant and negatively associated with AMP scores pre (r = −0.3, *p* = 0.01) and post (r = −0.2, *p* = 0.02), total stroke scores pre (r = −0.3, *p* = 0.01) and post (r = −0.3, *p* = 0.03) and total aquatic skill scores pre (r = −0.3, *p* = 0.01) and post (r = −0.2, *p* = 0.04). Similar results were found for fear of drowning, with it being significantly but negatively associated with AMP scores pre (r = −0.4, *p* < 0.05) and post (r = −0.3, *p* = 0.003), aquatic strokes scores pre (r = −0.4, *p* < 0.05) and post (r = −0.4, *p* < 0.05) and aquatic skill score pre (r = −0.4, *p* = 0.001) and post (r = −0.2, *p* = 0.02). Swimming opportunities have a positive significant relationship with AMP scores pre (r = 0.6, *p* < 0.05) and post (r = 0.4, *p* < 0.05), aquatic strokes pre (r = 0.6, *p* = 0.01) and post (r = 0.5, *p* < 0.05) and aquatic skills pre (r = 0.6, *p* = 0.001) and post (r = 0.5, *p* < 0.05). A negative significant relationship was found between AMP scores regarding both fear of water (*p* = 0.01) and fear of drowning (*p* < 0.05). A positive significant relationship was found between swimming opportunities and the AMP score (*p* = 0.001).

The mixed-model ANOVA showed a significant main effect in girls for pre- to post-data for seven out of the eleven fundamental aquatic skills (*p* < 0.05), with sculling feet first, submerge, floating and treading water having no significant effects (*p* > 0.05) (Table 1). Boys were found to have significant effects for pre- to post-data for seven out of eleven fundamental aquatic skills (*p* < 0.05), with submerge, floating, treading water and aquatic jump having non-significant changes over the 6-week period (*p* > 0.05) (Table 1). A main effect was found for both time points (pre to post) and interventions (control and intervention groups) for girls for the front crawl and breaststroke (*p* < 0.05), with submerge and floating skills having a significant main effect between the control and intervention groups (*p* < 0.05) (Table 1). A main effect was found for both time points (pre to post) and interventions (control and intervention groups for boys for breaststroke (*p* < 0.05), with floating skills having a significant main effect between the control and intervention groups (*p* < 0.05) (Table 1).

## 4. Discussion

This study is the first to examine the effects of an aquatic-based intervention on both a product and process-orientated assessment of FMS in children, investigating a 6-week period (pre to post) intervention looking at the effects it has on motor competence. This study was successful in implementing an aquatic intervention to distinguish the impact that a swimming program has in primary education. Showing an aquatic intervention significantly increases both aquatic motor competence and general motor competence. This study supports the affirmation that swimming embedded within a PE program enhances children’s FMS [33]. This study is making a novel contribution to the literature, although the links between the aquatic environment and motor competence have previously been investigated. To the authors’ knowledge, no research has explored the efficiency of this. This study also successfully achieved its second aim of investigating the links between an aquatic performance in relation to the fear of the water/ swimming and swimming opportunities.

The result from this study shows that both the intervention and control groups had significant increases in both general and aquatic motor competencies pre- to post-intervention. This study is the first to find that by replacing one PE lesson with a swimming lesson/aquatic intervention, there is a substantial increase in FMS. This further develops on the prior work, suggesting that school-based interventions enhance both the process [46] and product assessment of FMS [47]. The result from this study supports prior motor competence interventions where children who took part in non-structured/planned instruction increased competence in FMS [47,48]. This study advanced the previous works with aquatic programs, and although it was focussed on FMS development, it was anchored in a specific sport embedded within the national curriculum, other than a generic program as per prior work [16,17,18].

The framework within the intervention itself consisted of coaching cues, progressive practices, warmup, main activity and contrasting activities as per the learn to swim program outlined by Swim England [49]. This study is novel by incorporating a structured aquatic intervention embedded within the national curriculum enabling children to obtain key developments in FMS. Learning key aquatic skills and strokes and enhancing aquatic movement skills will, in turn, strengthen movement on dryland. Children who participated in this study, who were in either the intervention or control groups, increased their general and aquatic motor competence from pre- to post-data collection. This shows compelling evidence that implementing and maintaining any form of structured swimming program within a primary school PE setting will have significant improvements on a child’s motor development.

Through obtaining PA data, it was evident that children with any prior experience in swimming programs (afterschool lessons, leisure swimming, etc.) demonstrated optimised motor development on several gross motor skill tests. Implementing the fear of drowning and swimming opportunities questionnaire indicated the number of swimming opportunities an individual obtains and has positive significant correlations with aquatic motor competence. Individuals with higher fears of water and drowning achieved lower AMP scores. Individuals with more swimming opportunities achieved higher AMP scores. A range of aspects can influence a child’s ability to swim; some of these factors have been investigated previously, and the barriers that were highlighted include pool accessibility; fear of drowning; parents who fear the water discouraging their children from learning to swim; and many more [50]. This study shows that implementing an aquatic intervention significantly increases swimming opportunities for primary school-aged children, including those who have structured lessons within a physical education environment; therefore, in turn, this decreases both the fear of water and drowning. Implementing an aquatic intervention improves confidence and efficacy in the water, which was highlighted through the improvements that were observed in aquatic performance. Consequently, by the end of the intervention phase, with performance in the aquatic intervention increasing, a decrease in the fear of water and drowning through the implementation of water confidence practices was observed.

Another aspect of the national curriculum is for individuals to be able to perform self-rescue, developing a range of water safety skills [6]. This is a vital aspect of the national curriculum as educating individuals on water safety can save lives. A considerable amount of work has been directed towards educating the public about swimming and water safety to reduce the risk of drowning fatalities [51]. To best prepare children for safe aquatic participation is to provide the key skills and knowledge to lead a lifelong safe interaction with water [52]. Many studies regarding water safety and drowning rates are focussed abroad, with few studies being carried out in the United Kingdom [53]. In the United Kingdom, drowning is a leading cause of accidental and intentional death [54]. This study has demonstrated increased aquatic motor competence in both groupings, whether placed in the intervention or control group. This study has shown that implementing an aquatic intervention which develops aquatic performance, in turn, will develop key water safety skills, hence increasing the chances of performing safe self-rescue techniques in the water as per the national curriculum guidelines. This intervention incorporates a range of aquatic skills and strokes which can be strategically implemented in different water-based emergency situations, thus improving the likelihood of a child getting out of a dangerous situation. Therefore, working towards critical national curriculum requirements not only for developmental reasons but for health and safety aspects surrounding the aquatic environment is vital.

This study found that learning to swim in a school setting contributes to optimised performance in a various range of motor skills and environments (within both dryland and aquatic environments) (Figure 4 and Figure 5). This study demonstrates that improving dryland movements results in subsequent improvements in the aquatic environment [21,33]. The findings from this study support the previous work by Pratt et al. [44], showing that swimming is effective in enhancing motor competence and FMS. As a result of this aquatic intervention, both AMP and TGMD-3 scores significantly increased. Therefore, this intervention was successful in developing FMS on dryland as well as in the water. All individuals who partook in this study had significantly more defined FMS in addition to improved performance on all strokes and skills based in the water post-aquatic intervention. There are several explanations for this. Whilst control groups continued with normal planned PE lessons which consisted of one swimming lesson per week (which was the already implemented program within their PE program), the intervention groups followed the structured learning to swim program [55], which was delivered by trained swimming instructors. As a consequence, the control groups also improved in motor competence as did the intervention groups. This study provides an in-depth insight into the links between aquatic skills, strokes, and locomotor and object control skills, and provides a comprehensive analysis between dryland and aquatic skills by developing movements in water which also develop skills on land. Consequently, it shows that children who follow a structured swimming program will progress in both aquatic and dryland skills, regardless of the program which they follow. This is due to the positive effects that the water environment has on an individual, and as the density of the water allows the human body to become buoyant [37]. The weight relief and ease of movement in the water allows for a safe exploration of strength and functional activity training and enables individuals to produce movements that they may not be capable of on land [56]. Water provides buoyancy, which facilitates a full range of movement and postural control due to the reduction in gravitational effects it has, as well as the support it has on the individual [55].

This study is unique, finding a significant main effect for both boys and girls and specific FMS over time for a pre- to post-aquatic intervention. Motor competences improved for nine out of ten skills for girls and eight out of ten for the boys. This indicates that individual dryland movement skills are significantly impacted by developing movements in the aquatic environment. This study found key associations shown between individual AMP and TGMD-3 skills (Table 1). There is an acknowledgment in the literature that FMS differs with regard to sex [1]; therefore, these potential differences between boys and girls were considered when examining the impact an aquatic intervention has on FMS. This study found that boys obtained higher TGMD-3 skills compared to girls in both pre- to post-intervention and control groups. Similar to [57,58], boys were more proficient compared to girls in total FMS scores. However, when analysing the two subcategories, boys were found to perform better regarding object control skills compared to girls. This falls in line with the previous research, where boys outperformed girls regarding object control skills [57,58]. This difference could be attributed to the differences in physical activity levels, as boys are seen to have higher PA rates compared to girls. Girls are less likely to participate in PA than boys during preschool, especially at moderate- to high-intensity PA levels [57]. Differences in exercise content may also contribute to gender differences in object control [57]. Girls in this study were found to perform better on locomotor skills compared to boys. This is in line with [57], who found a trend favouring girls with regard to locomotor skills. Girls outperforming boys on locomotor skills has previously been accounted for by the types of activities in which they participate, with girls more likely to participate in sports with a greater emphasis on locomotor skills, including gymnastics and dance [59]. Boys obtained higher AMP, stroke and aquatic skills compared to girls in both the pre- to post-intervention and control groups. This ties in with the previous research, where boys were seen to obtain faster swimming times compared to their female counterparts. However, there are no data until this study which show a sex difference in aquatic motor competence in primary school-aged children, as sex-related research is based upon secondary school-aged to adult populations [60].

This study is the first to implement an aquatic-based intervention within a primary school-aged population to examine the effects that aquatic motor competence has on dryland movement. The main findings from this study are the significant increases in dryland motor competence as a result of an aquatic intervention. This study differs from previous studies due to the significant increases being found in both the intervention and control groupings. The previous research around the motor domain found significant developments in only the intervention groupings. Duncan et al. [20] found that their shuttle time intervention enhanced FMS within the intervention groups post-10-week intervention, with a specific increase in object control skills. A range of other intervention studies also saw significant increases in motor competence scores when comparing the intervention and control groupings [61,62]. However, similar to this study, Kelly et al. [63] found that both the control and intervention groupings increased motor competence as a result of their school-based FMS intervention. Moreover, Kelly et al.’s [63] results differed from this study as they found their groupings to have similar mean FMS scores after the intervention phases; this was due to the control having a higher FMS score before the start of the intervention phase. In this study, FMS increased in both the intervention and control groups as a result of them both participating in some form of aquatic PE sessions, whether an aquatic intervention or a structured swimming lesson was in place of the PE lessons.

Many interventions have been implemented with the aim of impacting motor development; however, there are few studies that investigated exactly which interventions are better compared to others on a range of different variables. Out of these interventions, there have been variations within their duration, from short-term to longitudinal interventions starting from 6 weeks [64], 10 weeks [65], 12 weeks [66] and up until 15 weeks [67]. Logan et al. [16] determined that the duration of the intervention was not associated with FMS improvement. Logan et al. [16] explained that this could be due to a plateau in FMS competence after time or due to the monotony of the intervention itself causing disengagement. This study selected a 6-week intervention to fit the intervention within one school term, including both pre- and post-data collection with no interference with school holidays. Future research should investigate the effects of an aquatic intervention on motor development for a longer duration to determine a longitudinal dataset. From this, the long-term impact of the intervention dose can be calculated, which can offer better insights into the potential effects of an aquatic intervention’s duration on motor learning and development.

Another element of intervention studies to consider is intervention dosage. Van Capelle et al. [17] suggested that motor competence interventions should be applied more than three times per week for more than 30 min to show any improvements. This, however, goes against the national curriculum in England, which suggests that every child should participate in two 60 min PE lessons per week. However, Robinson, Palmer and Meehan [68] found that intervention dosage is not the agent of change responsible for motor performance, suggesting there are other elements to interventions that determine effectiveness. Robinson, Palmer and Meehan [68] also highlighted how participating in free play leads to no improvement in motor skills; therefore, it is important for children to obtain high-quality movement opportunities within their PE classes to support the development of their FMS. This study follows these processes by implementing a high-quality intervention/control within the aquatic domain. This intervention consists of one 60 min PE lesson per week. In addition to this, it implements a swimming lesson/intervention, hence meeting the national curriculum of England’s requirements by law.

There are of course limitations to this study. Currently, there are no standardised dosages or guidelines for interventions within the aquatic domain due to this study being the first to implement an aquatic intervention within a primary school setting. The intervention length and duration of 6 weeks were selected pragmatically with educational terms and each school’s programs being the determinant of intervention duration and lesson length. Future research should be directed towards a program with a longer duration to see whether swimming enhances motor development further in comparison to this study’s shorter duration. However, unfortunately, due to the structures of lessons within primary schools, swimming lessons within the curriculum only last for one term. Additionally, many schools have significant barriers to being able to deliver swimming within their PE program. Depending on the location of the primary school, there are struggles as to whether there is an easily accessible local swimming pool that does not take too much time out of their educational program, as well as whether they are affordable [69]. However, Swim England [69] eliminated these barriers by producing resource packs for primary schools with guidance on tackling the issues outlined above. The resource pack details how the use of sports premium, provided by the Department for Education [70], can be implemented to provide every child with the opportunity to learn how to swim, detailing to schools how they can utilise this premium package to provide their students with regular swimming lessons. Another key limitation of this study is the lack of a control within the control group, with each school that was selected to act as a control participating in structured swimming lessons due to the nature of their PE programs. Future research should be implemented to include a control group without swimming embedded within the PE program of the school to compare the findings between swimming and non-swimming schools. Furthermore, it should investigate whether no swimming has an impact on motor development post 6 weeks of testing. However, this study found this to be problematic as swimming is compulsory for all primary school PE programs. Therefore, having schools not participate in swimming will result in going against the national curriculum. This study supports Swim England in showing the positive impact that swimming has on children, and the true prominence of children learning to swim, due to how extensively it develops FMS. Therefore, it supports the importance of swimming within the national curriculum of England.

## 5. Conclusions

To our knowledge, no studies have examined the impact of an aquatic intervention on motor competence. Through investigating the impact that a 6-week aquatic intervention has on both AMP and TGMD-3 total and sub scores, it can be concluded that focusing one PE lesson on swimming and aquatic development (one session per week for 6 weeks or more) will have a positive effect on dryland movements, which are key in the national curriculum of England. This study found significant improvements in both swimming skills and strokes, which has a positive impact on a child’s motor development. This study found both groups (intervention and control) to show significant improvements in both aquatic motor competence and general motor competence, thus supporting previous research; providing further evidence of the importance of swimming in the national curriculum; providing an advanced analysis between general and aquatic motor competence skills; and finding key associations between individual skills in both environments. The fear of drowning and fear of water has a considerable impact on a child’s motor development. Children with a lack of swimming opportunities are seen to have significantly poorer motor competence compared to those who are exposed to regular swimming opportunities. Due to the findings of this study, it can be concluded that an implementation of an aquatic intervention would be more beneficial to a child’s motor development than implementing a dryland intervention. Future research should include a control group from primary schools based with a non-swimming background. This will allow for a further investigation into the impact of a control group that does not partake in swimming. Therefore, this can provide direct links between swimming and non-swimming schools.

## Figures and Tables

**Table 1:** Descriptive data (mean ± SD) for key aquatic and dryland fundamental movements pre to post 6-week aquatic intervention for boys, girls, intervention and control groups.

	Intervention												Control											
	Girls						Boys						Girls						Boys					
	Pre			Post			Pre			Post			Pre			Post			Pre			Post		
	Mean	SD	95% CI	Mean	SD	95% CI	Mean	SD	95% CI	Mean	SD	95% CI	Mean	SD	95% CI	Mean	SD	95% CI	Mean	SD	95% CI	Mean	SD	95% CI
TGMD-2	40.1	1.4	37.3–42.9	51.1	1.6	47.6–54.5	46.6	2.9	40.5–52.6	57	3.1	50.6–63.5	38.6	1.6	35.3–41.8	52.5	2.1	48.1–56.9	42	2.6	36.5–47.4	58.5	3.8	50.3–66.6
Locomotor	23	0.7	21.5–24.5	28.9	0.8	27.2–30.5	22.6	1.5	19.6–25.7	28.3	1.4	25.3–31.3	23.9	0.9	22–25.8	28.9	0.7	27.4–30.5	21.2	1.4	18.2–24.2	30.1	1.2	27.4–32.7
Skip	3.8	0.2	3.3–4.3	4.7	0.3	4.2–5.2	3.8	0.3	3.1–4.5	47	0.4	3.9–5.5	3.7	0.2	3.4–4.1	4.6	0.2	4.1–5.1	3	0.4	2.2–3.9	5.1	0.2	4.6–5.5
Gallop	3.5	0.4	2.6–4.4	5.6	0.5	4.5–6.6	3	0.5	2–4	5.2	0.5	4.1–6.3	4.6	0.4	3.9–5.4	6.6	0.2	6.2–7	3.9	0.5	2.9–3.9	6.7	0.4	5.9–7.6
Hop	3.7	0.3	3–4.4	5	0.3	4.3–5.7	4.1	0.4	3.4–4.8	5	0.3	4.5–5.6	3.2	0.3	2.6–3.8	4.7	0.3	4.1–5.3	3.1	0.4	2.3–3.9	5.4	0.4	4.5 -6.2
Run	6.0	0.3	5.5–6.6	7.1	0.3	6.5–7.6	6.0	0.3	5.3–6.7	7	0.3	6.3–7.6	6.2	0.3	5.6–6.8	7.2	0.2	6.7–7.7	5.9	0.4	5–6.7	7.1	0.3	6.4–7.8
Jump	6.2	0.3	5.5–6.8	6.6	0.3	5.9–7.2	5.6	0.5	4.6–6.6	6.4	0.4	5.6–7.2	5.4	0.3	4.9–6	5.8	0.3	5.3–6.4	5.6	0.3	5–6.2	5.8	0.5	4.9–6.8
Object Control	17.2	1.1	14.8–19.5	22.3	1.1	19.9–24.7	23.9	1.7	20.3–27.5	28.7	1.9	24.9–32.6	14.6	1	12.7–16.6	23.6	1.6	20.3–26.8	20.8	1.8	17–24.5	28.4	3	22.1–34.7
Strike	4.5	0.3	3.9–5.1	4.3	0.5	3.2–5.4	5.4	0.6	4.2–6.7	6.3	0.5	5.2–7.4	3.3	0.3	2.7–4	5	0.6	3.9–6.2	5.1	0.5	4–6.2	6.1	0.7	4.5–7.6
Bounce	2.3	0.4	1.6–3	3.7	0.5	2.7–4.7	3.5	0.4	2.7–4.3	4.8	0.5	3.7–5.9	3.1	0.5	2.1–4.1	3.7	0.5	2.7–4.7	2.9	0.6	1.7–4.1	3.5	0.6	2.2–4.8
Catch	3.7	0.3	3.0–4.3	4.7	0.3	4.1–5.3	3.4	0.3	2.7–4.1	4.7	0.4	3.9–5.6	2.8	0.3	2.3–3.4	4.4	0.3	3.7–5.	3.2	0.3	2.5–4	4.4	0.5	3.3–5.4
Kick	2.7	0.4	1.9–3.5	3.2	0.4	2.4–3.9	4.9	0.5	3.7–6.0	5.3	0.6	4.2–6.5	1.3	0.2	0.8–1.7	2.8	0.4	2.1–3.6	3.2	0.5	2.1–4.3	4.9	0.7	3.4–6.4
Throw	1.8	0.3	1.2–2.5	2.6	0.3	2–3.2	3.9	0.5	2.8–4.9	3.5	0.6	2.3–4.7	1.2	0.3	0.6–1.7	2.4	0.5	1.4–3.3	2.4	0.5	1.4–3.4	4.5	0.8	2.8–6.3
Roll	2.2	0.4	1.3 -3.1	3.9	0.5	2.9–4.8	2.4	0.4	1.5–3.2	4.5	0.6	3.3–5.7	3	0.4	2.2–3.8	5.2	0.5	4.3–6.2	3.9	0.5	2.8–5	5.1	0.7	3.6–6.7
AMP	42.4	6.1	29.2–55.6	49.8	7.4	34.3–65.3	57	5.3	46–68	75.2	5.3	64.1–86.2	29.8	6.1	17.4–42.2	48	6.4	34.8–61.1	36.8	8.2	19.6–53.9	58	9.7	37.4–78.6
Strokes	22.3	3.8	14.1–30.5	28.9	4.3	19.8–38	31.6	3.4	24.2–39	42.4	3.4	35.4–49.4	14.1	3.4	7.2–20.9	22.3	0.6	14.9–29.8	16.9	4.7	7–26.7	28.1	4.8	17.8–38.3
Front Crawl	7.7	0.9	5.7–9.7	9.0	1.2	6.4–11.6	9.7	1.0	7.7–11.7	14	1.1	11.7–16.4	5.1	0.9	3.2–7	8.7	1.1	6.3–11	6.2	1.5	3.1–9.2	10.7	1.6	7.3–14.1
Back Crawl	9.9	1.7	6.1–13.7	9.9	0.6	6.5–13.4	11.3	1.3	8.6–14	14.4	1	12.3 -16.6	4.4	0.2	1.9–6.9	8.1	0.3	5.4–10.7	6	1.8	2.3–9.7	10.8	1.7	7.2–14.5
Breaststroke	4.7	1.6	1.2–8.2	10.5	0.9	6.6–14.4	10.6	1.7	7.1–14.1	15.6	1.3	13–18.2	4.5	0.3	2–7.1	4.9	1.5	1.8–7.9	4.7	1.7	1.1–8.3	6.5	2	2.4–10.7
Aquatic Skills	20.1	2.6	14.3–25.8	21.1	3.2	14.4–27.8	25.4	2.0	21.4–29.5	32.9	2.3	28.1–37.7	15.7	2.7	10.2–21.3	25.6	3.2	19.1–32.1	19.9	3.6	12.4–27.4	29.9	5.2	19–40.9
Glide	5.6	0.9	3.7–7.5	5.9	0.9	4–7.8	6.7	0.6	5.6–7.9	8.5	0.6	7.1–9.8	4.0	0.9	2.2–5.8	7.8	2.1	3.6–12	5.2	1	3–7.4	7	1	4.8–9.2
Scull Feet-first	0.2	0.2	0.2–0.5	0.3	0.2	−0.1–0.7	1.2	0.5	0.2–2.2	1.9	0.5	0.9–3				0.8	0.5	-0.1–1.7				2.5	1	0.3–4.6
Scull Head-first	0.9	0.8	-0.8–2.6	3.3	1.0	1.1–5.4	2.2	0.7	0.8 -3.6	4.2	0.8	2.5–5.9	2.1	0.7	0.7–3.5	3.6	0.9	1.8–5.3	2.3	0.9	0.3–4.3	4.7	1.1	2.3 -7.1
Tuck	0.2	0.2	-0.3–0.7	1.8	0.8	0.2–3.5				3.7	0.9	1.8–5.5	1.5	0.6	0.2–2.9	3.4	0.9	1.6–5.1	3	1.1	0.8–5.2	4.1	1.2	1.7–6.6
Submerge	3.4	0.3	2.6–4.1	2	0.6	0.8–3.2	3.6	0.2	3.14–4.1	3.2	0.4	2.4–4.1	1.8	0.6	1.1–2.5	2.7	0.3	2.1–3.3	2.3	0.4	1.4–3.1	2.6	0.4	1.7–3.5
Floating	6.0	0.7	4.5–7.5	5.1	0.7	3.7–6.5	5.8	0.4	5–6.5	5.4	0.5	4.3–6.4	2.6	0.5	1.7–3.6	5	0.6	3.9–6.2	2.8	0.7	1.4–4.2	3.5	0.7	2–5.1
Treading Water	1.2	0.8	−0.6–3	0.8	0.6	−0.4–2.1	2.3	0.7	0.9–3.8	3	0.8	1.4–4.6	1	0.5	0.3–2.0	1.7	0.6	0.4–3.0	2	0.8	0.3–3.7	2.4	0.9	0.4–4.3
Aquatic Jump	2.8	0.5	1.7–3.8	2.1	0.6	0.9–3.3	3.7	0.3	3–4.4	3.5	0.5	2.6–4.4	2.6	0.3	2.0–3.3	3.6	0.4	2.7–4.5	2.4	0.3	1.8–2.9	3.2	0.6	1.84–4.5

## Data Availability

Data are contained within the article.
